# A novel approach to completely alleviate peripheral neuropathic pain in human patients: insights from preclinical data

**DOI:** 10.3389/fnana.2024.1523095

**Published:** 2025-01-07

**Authors:** Safa Shehab, Mohammad I. K. Hamad, Bright Starling Emerald

**Affiliations:** Department of Anatomy, College of Medicine and Health Sciences, United Arab Emirates University, Al Ain, United Arab Emirates

**Keywords:** neuropathic pain, nerve injury, pain treatment, TRPV1, resiniferatoxin (RTX)

## Abstract

Neuropathic pain is a pervasive health concern worldwide, posing significant challenges to both clinicians and neuroscientists. While acute pain serves as a warning signal for potential tissue damage, neuropathic pain represents a chronic pathological condition resulting from injury or disease affecting sensory pathways of the nervous system. Neuropathic pain is characterized by long-lasting ipsilateral hyperalgesia (increased sensitivity to pain), allodynia (pain sensation in response to stimuli that are not normally painful), and spontaneous unprovoked pain. Current treatments for neuropathic pain are generally inadequate, and prevention remains elusive. In this review, we provide an overview of current treatments, their limitations, and a discussion on the potential of capsaicin and its analog, resiniferatoxin (RTX), for complete alleviation of nerve injury-induced neuropathic pain. In an animal model of neuropathic pain where the fifth lumbar (L5) spinal nerve is unilaterally ligated and cut, resulting in ipsilateral hyperalgesia, allodynia, and spontaneous pain akin to human neuropathic pain. The application of capsaicin or RTX to the adjacent uninjured L3 and L4 nerves completely alleviated and prevented mechanical and thermal hyperalgesia following the L5 nerve injury. The effects of this treatment were specific to unmyelinated fibers (responsible for pain sensation), while thick myelinated nerve fibers (responsible for touch and mechanoreceptor sensations) remained intact. Here, we propose to translate these promising preclinical results into effective therapeutic interventions in humans by direct application of capsaicin or RTX to adjacent uninjured nerves in patients who suffer from neuropathic pain due to peripheral nerve injury, following surgical interventions, diabetic neuropathy, trauma, vertebral disc herniation, nerve entrapment, ischemia, postherpetic lesion, and spinal cord injury.

## Introduction

The suffering associated with chronic pain remains one of the most challenging health issues in contemporary medicine. Acute physiological pain is an important alarm signal to alert us of potential injuries. However, neuropathic pain is a chronic pathological condition that develops subsequent to a lesion or disease affecting the somatosensory nervous system (Campbell and Meyer, 2006). Tissue/nerve injury, nerve compression, diabetes mellitus, inflammation, infections (e.g., herpes zoster), malignancies and autoimmune diseases can all result in neuropathic pain. The most common clinical presentations of neuropathic pain are: (1) long-lasting ipsilateral *hyperalgesia* (increased sensitivity to pain sensation); (2) *allodynia* (Pain due to a stimulus that does not normally provoke pain induced by light touch, brushing and stroking the skin, gentle pressure application to the body, warm or cold stimuli applied to the skin) and (3) *spontaneous unprovoked pain* (Campbell and Meyer, 2006). Neuropathic pain, therefore, has a significant and profound negative impact on patients’ quality of life, encompassing their social, economic, and psychological well-being.

The prevalence of neuropathic pain in the general population is alarmingly high, with estimates ranging from approximately 1% to as much as 7–10%. This incidence increases significantly when considering conditions such as diabetes, herpes zoster/shingles, and post-surgical pain (Bates et al., 2019). Despite advances in understanding the complex neurobiology of pain, effectively treating neuropathic pain remains a challenge, as many patients still experience insufficient relief (Cruccu and Truini, 2017; Finnerup, 2019; van Velzen et al., 2020; Finnerup et al., 2021; Shinu et al., 2022).

## A brief synopsis of the current treatments available for neuropathic pain and their associated limitations

### Pharmacological treatments

Pharmacological treatment is typically the first line of management of neuropathic pain. Various classes of drugs, including tricyclic antidepressants (amitriptyline and nortriptyline), serotonin-norepinephrine reuptake Inhibitors (duloxetine and venlafaxine), N-methyl-D-aspartate (NMDA) Receptor antagonists (ketamine), opioid analgesics (tramadol and oxycodone) and anticonvulsants/calcium channel α2-*δ* ligands (Gabapentin, pregabalin and Carbamazepine) are commonly used (Finnerup, 2019).

#### Limitations

These drugs are usually used only for short periods (not exceeding 4–8 weeks). To achieve sufficient pain relief, high doses of the medications are often needed. This can invoke many undesirable and limiting side effects (Baron et al., 2010). The problem is compounded in up to 45% of cases where patients are treated with two or more drugs (Simopoulos et al., 2008). Pharmacological treatments are usually terminated because they provide inadequate pain-relief, unacceptable side effects, or a combination of both (Baron et al., 2010; Finnerup et al., 2015, 2021; Finnerup, 2019).

### Ion channels

Ion channels are essential in defining the fundamental properties of cell membranes. Neurons use various ion channels such as sodium (Na^+^), calcium (Ca^2+^), potassium (K^+^) and chloride (Cl^−^), which regulate electrical potentials and affect the neurotransmission in the central as well as the peripheral nervous system. Therefore, it is not surprising to find that ion channels play a significant role in the transmission and modification of acute pain sensation and might contribute to the development of neuropathic pain.

Nine types of voltage-gated Na^+^ channels (Nav1.1 to Nav1.9) have been identified (Waxman and Zamponi, 2014). Of these, Nav1.7, Nav1.8, and Nav1.9 are prevalent in the adult dorsal root ganglion (DRG) neurons, suggesting their possible involvement in the sensitization of sensory neurons in neuropathic pain (Dib-Hajj et al., 2015; Stevens and Stephens, 2018; Bennett et al., 2019; McDermott et al., 2019). Consequently, Nav1.8 blocker (PF-04531083, Pfizer) and Nav1.7 blocker (CNV1014802, known as raxatrigine, Convergence Pharmaceuticals) are under clinical trials. The former is being tested for its potential in treating diabetic neuropathy, while the latter is being evaluated for its efficacy in treating trigeminal neuralgia and lumbosacral radiculopathy.

Ca^2+^ channels are crucial in regulating sensory functions related to the transduction, transmission, processing, and modulation of pain signals (Yaksh, 2006). Three subtypes based on the pore-forming α1 subunit, calcium voltage-gated channel subunit alpha G (Cav3.1), calcium voltage-gated channel subunit slpha1 H (Cav3.2), and calcium voltage-gated channel subunit alpha1 I (Cav3.3) have been identified (Catterall et al., 2005; Iftinca, 2011) and have also been shown to play important roles in neuropathic pain (Luo et al., 2001; Feng et al., 2019).

K^+^ channels are essential for regulating neuronal excitability. Various subclasses of voltage-gated potassium channels (Kv), such as Kv1.1, 3.3, 3.4, 4.1, 4.2, 4.3, and 9.1, have been expressed in sensory neurons. Research suggests that dysfunction in any of these channels could potentially contribute to the onset of neuropathic pain (Rasband et al., 2001; Chien et al., 2007; Tsantoulas et al., 2012; Tsantoulas and McMahon, 2014; Smith, 2020; Kanda et al., 2021).

Table 1 summarizes various studies demonstrating the involvement of Na^+^, Ca^2+^, and K^+^ channels in neuropathic pain. The methods used to alleviate neuropathic pain in these studies typically include pharmacological approaches, such as channel blockers and inhibitors. These methods provide insights into how altering the function of Na^+^, Ca^2+^, and K^+^ channels can affect neuropathic pain states, potentially leading to the development of novel therapeutic approaches.

**Table 1:** This table presents an overview of studies examining Na+, K+, Ca2+ ion channel modulation for neuropathic pain treatment across various animal models.

Ion channel	Neuropathic pain models	Treatment	References
Na_V_1.7	Intraplantar injection of the scorpion” *Odonthobuthus doriae”* toxin OD1, NaV1.7 KO model, Freund’s Complete Adjuvant (FCA)-induced inflammation	Na_V_1.7 inhibitor. μ-Theraphotoxin Pn3a (*μ*-*TRTX*-*Pn3a*), a three-disulphide bridged, 35 amino acid peptide, a peptide isolated from venom of the South American tarantula *Pamphobeteus nigricolor*.	Deuis et al. (2017)
	Small fiber neuropathy (SFN)	Na channel blocker. Vixotrigine (BIIB074), currently completed phase II clinical trial	Biogen (2021)
Na_V_1.8	L5–L6 nerve injury SNL	Sodium Na_V_1.8 blocker. A-803467 [5-(4-chlorophenyl-N-(3,5-dimethoxyphenyl) furan-2-carboxamide].	Jarvis et al. (2007)
	L5 nerve injury (SNL) and inflammatory neuropathic pain	Sodium Na_V_1.8 blocker PF-01247324 [6-amino-5-(2,3,5-trichloro-phenyl)-pyridine2-carboxylic acid methylamide].	Payne et al. (2015)
	Bunionectomy	VX-548 (Phase 2)	Clinicaltrials.gov 2021
Na_V_1.9	Chronic compression of DRG	Intervertebral foramen plerixafor injection (IVFP)	Yang et al. (2021)
	Rat models of thermal (Hargreaves test, Hotplate test and Tail-flick test) and chemical (formalin injection) nociception.	JNJ63955918, Nav1.7 blocking peptide.	Flinspach et al. (2017)
K_V_1.3	Spared nerve injury model (SNI)	PAP-1 (5-(4-Phenoxybutoxy) psoralen)	Yuan et al. (2023)
	Rat models of rheumatoid Arthritis	ShK-235 (LrS235) Kv1.3 potassium blocker	Wang et al. (2023)
K_V_4.3	Trigeminal neuropathic pain in male rats following infraorbital nerve chronic constrictive injury.	Phrixotoxin-2 (spider venom), Kv4.3 channel inhibitor	Kanda et al. (2021)
Ca_V_2.2	Complete Freund’s adjuvant model of inflammatory pain.	ω-conotoxin inhibitors CVID, MVIIA and GVIA (C9915) (these do not have a full form; they are class of drugs)	Rycroft et al. (2007) and Pitake et al. (2019)
	(a) Paw incision model of postoperative pain (b) Spinal nerve ligation model of neuropathic pain	*IPPQ* *Chemical name: 2-(3,5-dimethylisoxazol-4-yl)-N-((4-((3-phenylpropyl) amino) quinazolin-2-yl) methyl) acetamide*	Khanna et al. (2019)
	Refractory chronic pain	*Prialt* (ziconotide intrathecal infusion) *(FDA approved)*	Lynch et al. (2006)
	L5-L6 injury Spinal Nerve Ligation	N-triazole oxindole (*TROX-1*)	Patel et al. (2015)
	Partial sciatic nerve ligation (PSNL)	Icariin (ICA)	Pokkula and Thakur (2021)
Ca_V_3.2	PSNL	Cav3.2 T-type channel blocker. KYS-05090S (a member of the 3,4-dihydroquinazoline class)	M’Dahoma et al. (2016)
	PSNL	TTA-A2 (a derivative of 4-aminomethyl-4-fluoropiperdine), NiCl_2_ and mibefradil	Feng et al. (2019)
Ca_V_3.2	Mouse models of inflammatory and neuropathic pain	ABT-639 Cav3 inhibitor	Antunes et al. (2024)

#### Limitations

Sodium channel blockers are associated with adverse effects like double vision, delirium, and somnolence (Shinu et al., 2022). Despite over 15 years of extensive research leading to the development of several highly potent and selective NaV1.7 channel inhibitors, there have been limited instances where these inhibitors have effectively provided pain relief in preclinical models or human clinical trials (Eagles et al., 2022).

Currently, no analgesic medications alter pain signaling *via* potassium channels (Shinu et al., 2022).

The translation of these studies presents difficulties since animal models do not accurately represent complex pain states (Smith, 2023), and pain cannot be resolved through a single target due to its multifactorial nature.

Further research could advance the development of innovative and effective pain-relieving medications.

### Interventional therapies

Epidural steroid injections have been used in persistent radiculopathy due to herniated lumbar disc (Zhang et al., 2024).

#### Limitations

In practice, epidural injections may generally provide limited short-term relief, and a recent review concludes that there is insufficient evidence to support the assertion that epidural steroid injections produce significant effects compared to saline (de Bruijn et al., 2021).

### Neurostimulation

Recent studies have explored direct stimulation techniques for treating neuropathic pain. These methods involve using a compact electrical device to administer stimulation pulses with different intensities and frequencies directly to the spinal cord or dorsal root ganglia (Joosten and Franken, 2020; Abd-Elsayed et al., 2024; Sammartino et al., 2024).

Pulsed radiofrequency (PRF) is a nondestructive radiofrequency technique that passes an electrical field across the nerve, thereby exerting neuromodulatory effects on synaptic transmission. In this type of treatment radiofrequency generator is used to deliver stimulation through a needle introduced through a cannula aimed at the corresponding DRG (Simopoulos et al., 2008; Horan et al., 2021).

Radiofrequency (RF) denervation is a destructive technique where heat ablates the offending nerve. Lesioning of the primary neurons in the dorsal root ganglion can be used as an option for the treatment of radiculopathy (Walsh et al., 2022).

#### Limitations

In addition to requiring specialized skills and surgical procedures, spinal cord stimulation therapy faces significant challenges, such as a decline in efficacy over time and stimulation habituation (Sammartino et al., 2024). This type of treatment may lead to significant complications, including therapy habituation, electrode damage and disconnection, and unpleasant sensations of paresthesia (Joosten and Franken, 2020; Shinu et al., 2022). Furthermore, there is a need for clinical trials that are not sponsored by industry (Sammartino et al., 2024).

DRG stimulation requires specialized surgical procedures and the expertise of experienced neurosurgeons. Although it can be effective for some conditions, in majority of patients the beneficial effects of this treatment were lost after 8 months (Simopoulos et al., 2008).

Additionally, the implantation of electrodes into sensory pathways carries the risk of causing necrotic damage, potentially exacerbating neuropathology. The evidence supporting the effectiveness of direct dorsal root ganglion stimulation for relieving neuropathic pain remains, at best, inconclusive (Horan et al., 2021).

Furthermore, evidence for how effective RF denervation of the DRG for treating radiculopathy is limited (Bates et al., 2019).

### Repetitive transcranial magnetic stimulation (rTMS)

rTMS of the primary motor cortex (M1) is a noninvasive brain-stimulation method that has attracted attention as an alternative treatment for intractable neuropathic pain (Lefaucheur et al., 2014; Attal et al., 2021). This technique employs a brief, high-intensity magnetic field applied to the cerebral cortex to produce induced currents. It modifies the action potential of cortical nerve cells, depolarizes neurons in the targeted brain region, and results in neuroplastic changes.

#### Limitations

Despite its promising potential therapeutic effects, recent randomized controlled clinical studies have shown that rTMS does not significantly relieve neuropathic pain (Hosomi et al., 2020; Mori et al., 2024).

### Topical treatment with capsaicin

While the topical application of capsaicin (CAPS, trans-8-methyl-N-vanillyl-6-nonenamide) to the skin initially induces a burning pain sensation, this is followed by prolonged attenuation of pre-existing pain from the same region. It has therefore been used widely as a tool for producing and treating pain in clinical and preclinical studies. Either a low dose of CAPS cream (<1% applied 2–3 times per day for 6–8 weeks) or a higher dose (8% as a single application) have been used as topical treatments for neuropathic pain (Anand and Bley, 2011).

#### Limitations

Again, evidence for the effectiveness of topical CAPS is equivocal. A Cochrane Database review concluded that low concentrations of topical CAPS fared no better than placebo creams (Derry and Moore, 2012). However, a later systematic review (Derry et al., 2017) reported that high-concentration topical CAPS can be helpful for approximately 10% of neuropathic pain patients.

### Botulinum toxin-A

Botulinum toxin (BoNT) is a neurotoxic protein produced by the bacterium *Clostridium botulinum.* It is the causative agent of botulism, a form of food poisoning. Based on its known action in blocking the release of acetylcholine at neuromuscular junctions, BoNT/A has been used to treat a wide variety of conditions, including muscle spasticity associated with central nervous system (CNS) disorders (Charles, 2004). In addition, studies conducted in animals and humans have shown that BoNT/A can effectively treat some forms of neuropathic pain (Finnerup et al., 2015).

#### Limitations

Precise perineural, high-resolution, ultrasound-guided BoNT/A injection reduced pain intensity by ≥30% in approximately 60% of patients tested, with 40% failing to respond (Meyer-Frießem et al., 2019). Thus, evidence for the efficacy of this treatment is also equivocal.

The conclusion is that many of the current treatments for neuropathic pain provide incomplete relief to a variable subset of patients. There remains, therefore, a pressing unmet need for more effective treatments for neuropathy patients who suffer from this most debilitating chronic condition (Smith, 2023). Developing novel therapeutic interventions to control neuropathic pain is therefore critical (Finnerup et al., 2015; Finnerup, 2019; van Velzen et al., 2020; Smith, 2023).

This review aims to evaluate a promising treatment developed in our laboratory, as well as in others, for the control of neuropathic pain. Notably, we present a novel approach for treating and preventing neuropathic pain in animal models, with the goal of translating it to clinical applications for patients suffering from this debilitating condition. To fully comprehend the novel and innovative nature of this approach it is essential to have a fundamental understanding of the neurobiology of pain.

### The neurobiology of pain

**Figure 1:**
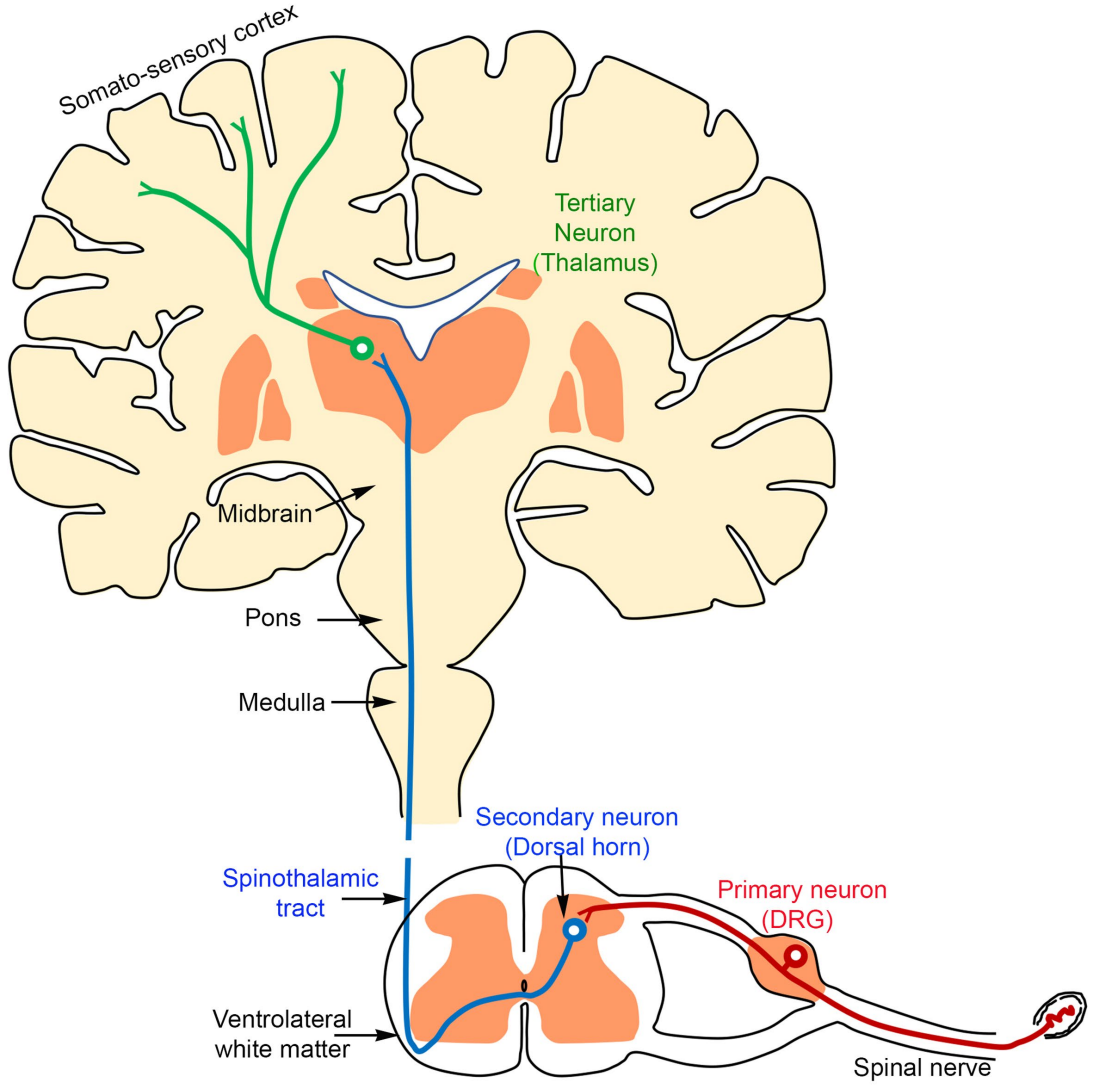
A schematic diagram showing that the transmission of the sensory information from the periphery reaches the brain through three sequentially connected neurons: primary (red, located in the DRG), secondary (blue, located in the dorsal horn of the spinal cord), and tertiary (green, located in the thalamus).

Pain is typically experienced when nociceptive (pain-related) information from peripheral sources, such as the skin, joints, bones, and viscera, is transmitted *via* the spinal cord to the brain. This process involves a sequence of at least three classes of neurons (primary, secondary, and tertiary) that relay pain signals to central processing centers in the brain, including the thalamus and somatosensory cortex:

- Primary afferents transmit pain sensations from the periphery to neurons in the spinal cord, located in the superficial layers of the dorsal horn.
- Secondary dorsal horn neurons project signals to the thalamus.
- Subsequently, tertiary thalamic neurons then project to the somatosensory cortex (Figure 1).

Our laboratory focuses on the critical neural processes between primary and secondary afferents in the dorsal horn of the spinal cord.

#### Role of neuronal circuits in the dorsal horn of the spinal cord in pain transmission

**Figure 2:**
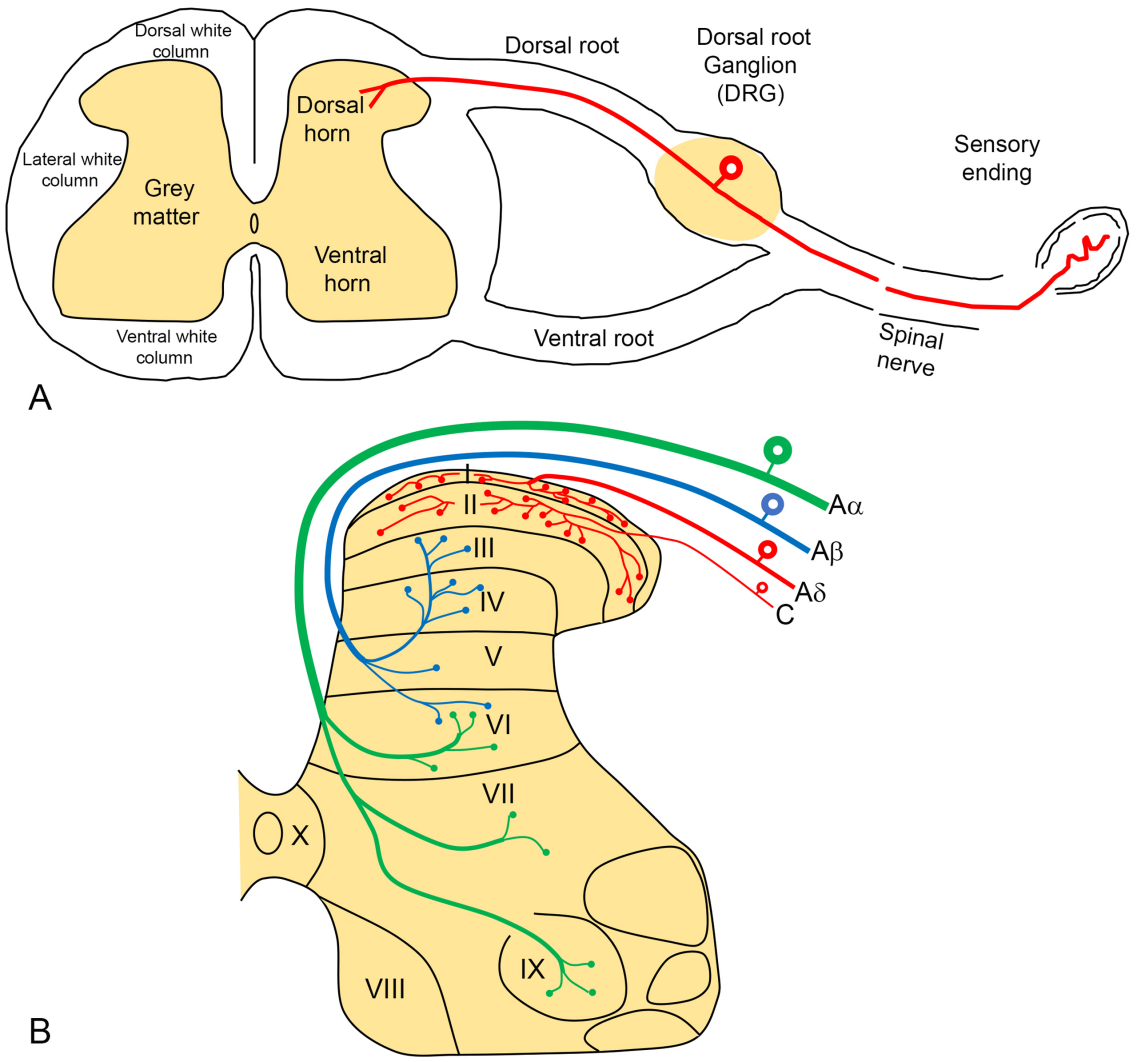
**(A)** A diagram illustrating a cross-section of the spinal cord, showing the formation of a peripheral spinal nerve. Pain sensory information from the periphery terminates in the dorsal horn of the spinal cord via the primary neurons, whose cell bodies are located in the dorsal root ganglion (DRG). **(B)** The gray matter of the spinal cord is organized into 10 laminae, with pain sensory fibers (red) terminating predominantly in the superficial laminae (I-II).

Examination of a cross-section of the spinal cord reveals a central H-shaped gray matter (consisting of mainly cell bodies) surrounded by white matter (nerve fibers) (Figure 2A). The gray matter is divided into dorsal (top) and ventral horns (bottom), which are also divided into 10 laminae (layers) (Figure 2B).

Laminae I-VI are located in the dorsal horn, with lamina I being the most dorsal (superficial) layer. Lamina I and II (together known as substantia gelatinosa) receive and modulate afferent pain information from the periphery (Figure 2B).

#### Primary afferent neurons

The cell bodies of primary afferent neurons, which transmit pain information from peripheral sites to the spinal cord, are located in the DRG (Figure 2A). These neurons are typically subdivided into small, medium and large cells characterized by a unique morphology that includes two arm-like processes: peripheral and central. The peripheral processes have sensory endings in the skin, joints and muscles, transmitting pain information to the cell bodies situated in the DRG. The central process extends from the cell body to the dorsal horn neurons in the spinal cord (Figure 2A). In this region, the branching of DRG terminals is highly organized (Figure 2B). Small and medium-sized neurons in the DRG (the red neurons in Figure 2B) give rise to the unmyelinated C and thin myelinated Aδ fibers associated with pain transmission. These fibers predominantly terminate in the superficial layers (laminae I and II) of the dorsal horn.

To study pain transmission, it is essential to identify pain-related neurons in the laboratory. The most commonly used procedures are those which detect neuron-specific intracellular substances known as neuropeptides, which can be identified histochemically. These include calcitonin gene-related peptide (CGRP), substance P (SP), the CAPS-sensitive ion channel receptor transient receptor potential vanilloid subfamily type 1 (TRPV1), and isolectin B4 (IB4). Experiments have demonstrated that these substances are localized in small-and medium-sized neurons in the DRG, which have central nerve terminals in laminae I and II of the dorsal horn (Shehab et al., 2004, 2015; Shehab, 2014; Javed et al., 2020, 2022). In contrast, they are absent in large DRG neurons that possess thick myelinated Aβ nerve fibers and predominantly terminate in the deeper layers (laminae III and IV and beyond) of the spinal cord (black neurons in Figure 2B; Javed et al., 2020). These larger DRG neurons are associated with the transmission of mechanoreceptor and touch information (Todd, 2010).

#### Capsaicin (CAPS)

The Nobel Prize for the year 2021 in Physiology and Medicine was awarded to Professors David Julius and Ardem Patapoutian in recognition of their discoveries of the receptors for temperature and touch. Professor Julius worked to identify the receptor for CAPS, an active component in hot red peppers that causes a painful burning sensation. They used CAPS to identify its endogenous receptor Transient Receptor Potential Vanilloid 1 (TRPV1), an ion channel activated by painful heat. The prize committee reported that this discovery “is being used to develop treatments for a wide range of disease conditions, including chronic pain.” This review proposes to translate the preclinical results using CAPS and its highly potent analog resiniferatoxin (RTX) from rodents to humans to provide a novel, clinically effective treatment of chronic neuropathic pain.

When administered directly onto peripheral nerves, CAPS has paradoxical dual actions. Topical application of capsaicin has been demonstrated to induce a burning sensation by activating the TRPV1 ion channel in human skin and mucous membranes. However, after this initial activation, the threshold for painful stimuli rises, resulting in desensitization (Jancśo et al., 1980; Szolcsányi, 2014). As a result, capsaicin and its highly potent analog, resiniferatoxin (RTX), have been extensively utilized in both clinical and preclinical research for pain management (Brown, 2016; Fattori et al., 2016; Moran and Szallasi, 2018; Sapio et al., 2018; Iadarola et al., 2021).

An early investigation reported that peripheral application of CAPS had been shown to produce selective thermal and chemical analgesia accompanied by the abolition of neurogenic inflammation in the skin area supplied by the treated nerve (Jancsó, 1992). Moreover, following the local administration of CAPS to the sciatic nerve, the neuropeptide SP was depleted in the skin, the DRG and the superficial layers of the dorsal horn of the spinal cord (Gamse, 1982).

CAPS blocks the transmission of pain information along the unmyelinated pain-related C-fibers of the small DRG neurons. This happens within minutes following perineural exposure to CAPS (33 mM ~ 1%). Larger A-fibers associated with tactile (touch) appear to exhibit greater resistance to this blocking action (Szolcsányi, 2014). This selectivity for pain transmission has been demonstrated by showing preserved responses to touch in CAPS-treated animals (Javed et al., 2020).

The mechanism of action of CAPS and its analog RTX is to operate through the TRPV1 receptors, identified by the recent Nobel laureates. These receptors are located on GRG neurons, which give rise to C-fibers with unmyelinated axons. Thus, perineural treatment of the sciatic nerve with 1% CAPS produced a rapid decrease in TRPV1 mRNA and protein expression in the DRG (Szigeti et al., 2012). This downregulation of TRPV1 in the DRG neurons was associated with reduced responses to thermal pain and a reduction in thermal hyperalgesia. These findings suggest that the analgesic action of CAPS operates, at least in part, through the TRPV1 receptor.

### Preclinical model for the suppression of neuropathic pain

Human experimental pain models, particularly those utilizing contemporary imaging techniques, have significantly contributed to our understanding of the neural systems that process pain. However, investigating the mechanisms by which pain information is processed within these systems typically requires invasive experimentation that cannot be conducted with human subjects. As a result, animal models are widely employed to study pain mechanisms, including those associated with neuropathic pain.

It is widely accepted that neuropathic pain represents a pathological response to injury within the somatosensory system (Campbell and Meyer, 2006). Consequently, most animal models of this condition involve experimental injury to peripheral nerves (Ossipov and Porreca, 2013). For instance, neuropathic pain in animals can be induced by constriction (ligation) and/or severance of nerves, which is a commonly used model (Ho Kim and Mo Chung, 1992). In this model, the fifth lumbar (L5) spinal nerve is ligated and cut, leading to the development of ipsilateral hyperalgesia, allodynia in the hind paw, and spontaneous pain in rats.

**Figure 3:**
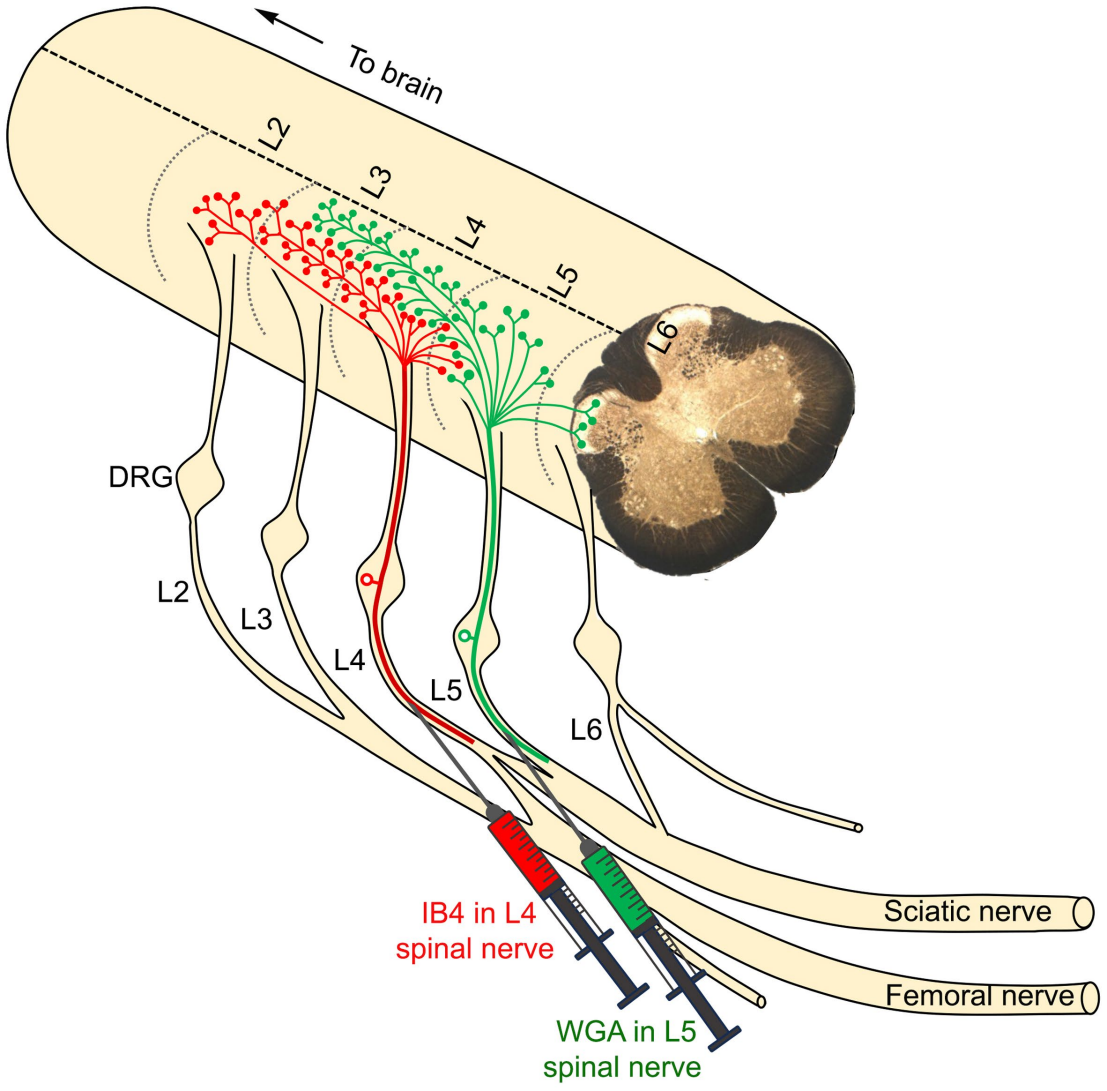
The primary afferents of two adjacent peripheral nerves were identified by two distinct tracers: IB4 (green in color), injected into the L4 nerve, and WGA (red in color), injected into the L5 nerve. The nerve terminations of the L4 primary afferents were observed in the dorsal horn of the corresponding L4 segment, extending into two rostral segments (L2 and L3) and one caudal segment (L5). Similarly, the nerve terminations of the L5 primary afferents were observed in the L5 segment, extending rostrally into the L3 and L4 segments and caudally into the L6 segment. Notably, there is significant overlap between the central terminals of the L4 and L5 spinal nerves within the dorsal horn of the L4 segment (Shehab et al., 2008).

Using this model, an important anatomical discovery has provided a critical foundation for the novel approach proposed in this review. It was found that unmyelinated pain-related primary afferents from adjacent spinal nerves intermingle as they terminate in the spinal dorsal horn (Shehab et al., 2008). This indicates that the spinal terminations of pain-related afferents from the L4 and L5 spinal nerves overlap in the dorsal horn at the L3-L5 spinal levels (Figure 3). This suggests that a single neuron in the dorsal horn of the spinal cord may receive pain-related afferent signals from two or more adjacent nerves (Shehab et al., 2008). The resulting hypothesis posits that this anatomical overlap of incoming pain information could contribute to the neuropathic pain that develops following injury to just a single afferent nerve (Shehab et al., 2008). Specifically, the idea is that the response of adjacent but uninjured primary afferents may play a critical role. This proposal shifted the prevailing focus from a widely accepted peripheral mechanism to a central one in the dorsal horn of the spinal cord.

**Figure 4:**
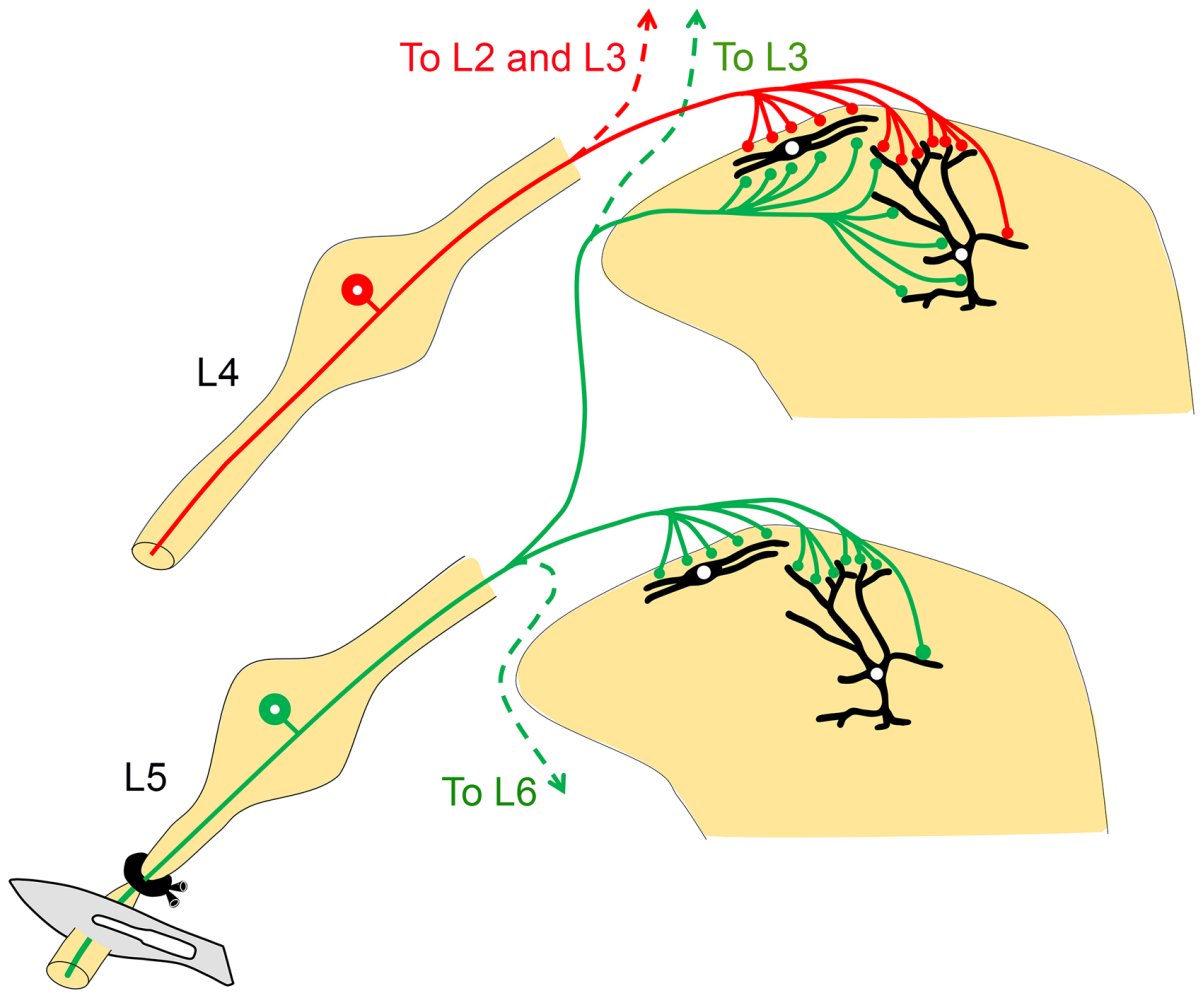
A schematic diagram illustrates how the intermingling of primary afferents from two adjacent peripheral nerves may explain the manifestations of neuropathic pain following peripheral nerve injury. Injury to the L5 nerve sensitizes the projection neurons (shown in black in the dorsal horn) in its corresponding spinal segment as well as in the two rostral segments (L3 and L4). Stimulation of the uninjured L4 nerve then leads to overactivation and hypersensitivity via the sensitized projection neurons in the L4 spinal segment, likely resulting in hyperalgesia (adapted from Shehab, 2014).

The next step in testing the hypothesis was to determine whether the ligation of one afferent nerve produced measurable changes in the neurochemistry of overlapping territories in the dorsal horn (Figure 3). The results indicated a significant downregulation of several markers (IB4, CGRP, and SP) and upregulation of others [vasoactive intestinal polypeptide (VIP), neuropeptide Y (NPY), and neurokinin-1 receptor (NK1r)]. Importantly, these changes occurred not only in the L5 segment where the ligated L5 nerve terminates, but also in the adjacent segments (L3, L4, and L6). These findings further support the notion that the observed changes in these neurochemical and neuroplastic markers may represent a critical substrate for the central sensitization observed in denervated regions of the dorsal horn (Shehab, 2014). Thus, sensitized projection neurons in the L4 segment, resulting from adjacent L5 denervation, may be responsible for the exaggerated responses to painful and tactile stimuli applied to regions of the skin innervated by the uninjured L4 nerve (Figure 4). This framework aligns with the requirement that hyperalgesia and allodynia necessitate an intact nerve to conduct noxious and tactile information from the hind paw skin to the spinal cord. Therefore, when the L5 nerve has been ligated, the adjacent L4 nerve is the most likely candidate to mediate the resulting neuropathic pain. Additional evidence arises from a subsequent anatomical investigation, in which the phosphorylation of extracellular regulated kinases (pERK) was used as a marker for pain activity. The findings demonstrated that hypersensitivity in the uninjured L4 spinal nerve significantly contributed to the development of hyperalgesia in the hind paw skin following L5 nerve injury in rats (Shehab et al., 2015).

**Figure 5:**
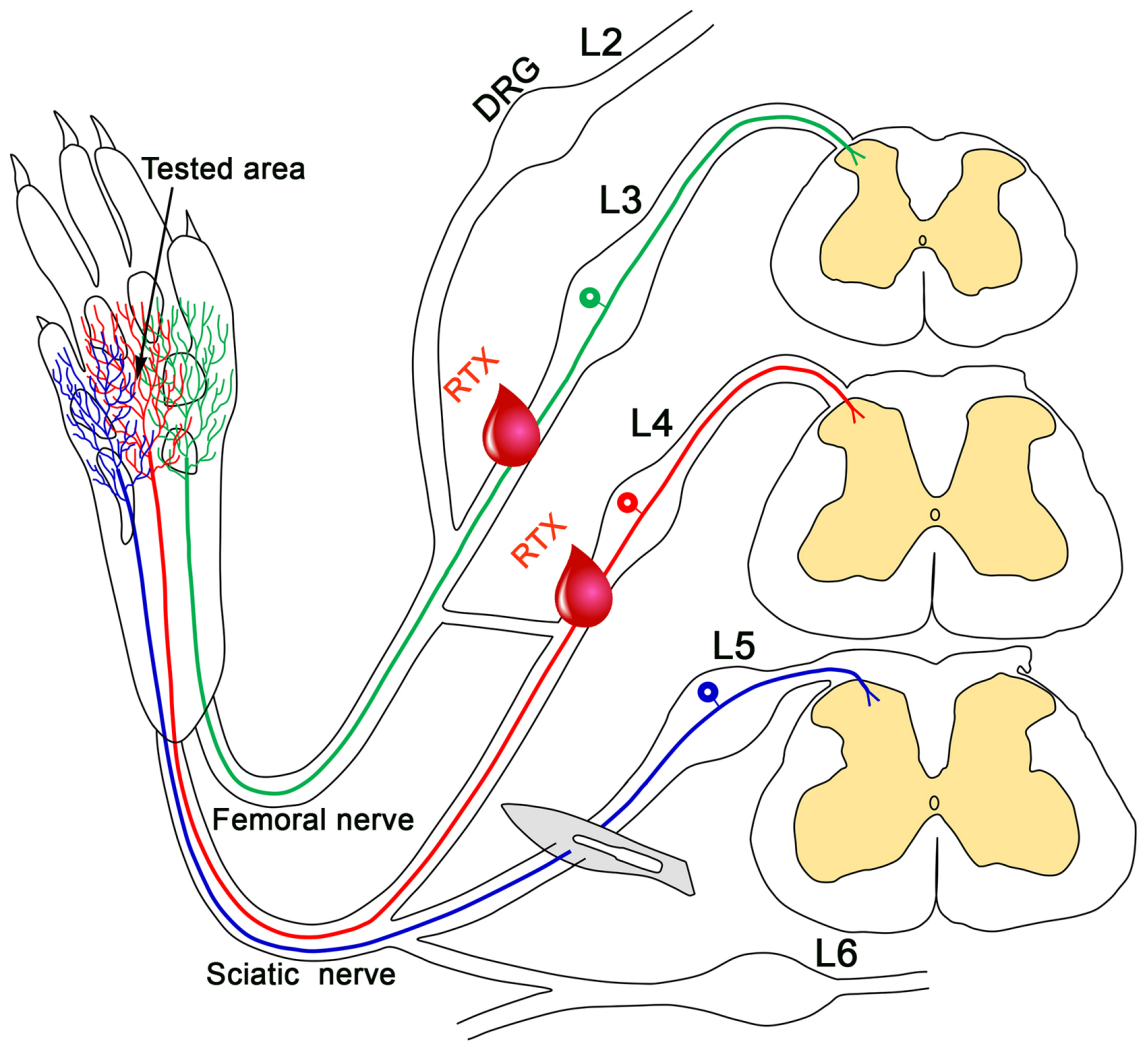
This schematic diagram illustrates how applying RTX to uninjured peripheral nerves alleviates nerve-injury-induced neuropathic pain. The plantar skin of the rat is innervated by the L3 nerve (green), which is supplied through the saphenous branch of the femoral nerve, as well as by the L4 (red) and L5 (blue) nerves, which are part of the sciatic nerve. The blue, red, and green labeled nerves on the skin indicate overlapping dermatomes of L3-L5 nerves in the plantar skin. Injury to the L5 nerve results in neuropathic pain in the plantar skin of the hind paw (tested area). The application of RTX to the uninjured L4 nerve significantly reduces this neuropathic pain. Notably, administering RTX to both the uninjured L3 and L4 nerves completely alleviates the neuropathic pain following L5 nerve injury.

To determine whether this is true, the final step was to establish whether the uninjured L4 nerve plays a critical role in mediating the neuropathic response following L5 injury. We utilized the selective pain-blocking properties of CAPS to inactivate the intact L4 nerve (with CAPS or RTX) following L5 nerve injury (Javed et al., 2020). The results demonstrated that the neuropathic pain induced by adjacent L5 nerve injury was significantly reduced. Compared to the contralateral side or vehicle treatment, applying both CAPS and RTX to the uninjured L4 nerve reliably decreased hypersensitivity to thermal and mechanical pain following L5 nerve injury (Javed et al., 2020). It was noted, however, that these treatments suppressed but did not completely abolish the increased sensitivity to pain following L5 nerve ligation. Based on previous anatomical findings showing substantial overlap (Shehab et al., 2008), both the L4 and L3 nerves were inactivated after L5 nerve ligation rather than just the L4 nerve (Figure 5). Over a period of 28 days, this treatment completely blocked measures of neuropathic pain (i.e., hypersensitivity to thermal and mechanical pain; Javed et al., 2020).

### Effects of perineural application of RTX to intact peripheral nerves on normal pain behavior

Although TRX treatment alleviates and prevents nerve-injury-induced neuropathic pain, its similar application in normal rats without nerve injury did not produce significant differences in the responses of animals to thermal and mechanical stimuli (Jang et al., 2007; Javed et al., 2020; Shehab et al., 2023).

To achieve complete relief and prevent the development of neuropathic pain symptoms following L5 nerve injury in rats, perineural application of RTX to both the L3 and L4 nerves was necessary (Javed et al., 2020; Shehab et al., 2023). Notably, when RTX was administered to the uninjured normal L3-L5 nerves, which innervate the plantar skin of the rat (Takahashi et al., 1994, 2002; Javed et al., 2020), no significant variation in withdrawal latency to thermal and mechanical stimuli was observed compared to the untreated hind paw (Javed et al., 2020). Similarly, an investigation revealed that applying capsaicin exclusively to the L4 nerve did not affect mechanical sensation (Jang et al., 2007). In another approach, the intrathecal administration of RTX in rats eliminated TRPV1 immunoreactivity in the dorsal horn of the spinal cord and prevented inflammatory hypersensitivity but had no effect on acute thermal and mechanical sensitivities (Bishnoi et al., 2011). These findings suggest that responses to thermal and mechanical stimuli remained within normal ranges for up to 28 days post-treatment (Javed et al., 2020). Additionally, other studies have reported that perineural application of RTX effectively inhibits inflammatory hypersensitivity while minimally impacting normal thermal and mechanical sensations (Neubert et al., 2008).

This is supported by the fact that TRPV1-deficient mice exhibited typical reactions to acute noxious stimuli but failed to develop thermal hyperalgesia induced by carrageenan (Davis et al., 2000; Woodbury et al., 2004). Interestingly, these mice displayed only slight behavioral responses to intense radiant heat or harmful temperatures exceeding 50–52°C (Caterina et al., 1999; Marics et al., 2014). The limited impact on normal sensation suggests that the role of TRPV1 may be more critical in chronic pain conditions than in the perception of acute pain stimuli (Woodbury et al., 2004; Javed et al., 2020). Furthermore, these findings underscore the potential dissociation between the transmission of normal physiological pain and the abnormal hyperalgesia observed in neuropathic pain. From a therapeutic perspective, leveraging this dissociation may prove advantageous in the effective management of chronic pathological neuropathic pain (Javed et al., 2020; Shehab et al., 2023).

### Mechanisms of action of CAPS and RTX in completely relieving neuropathic pain

Several mechanisms are required to explain how the application of RTX to the uninjured L4 nerve significantly reduces neuropathic pain following L5 nerve injury (Javed et al., 2020, 2022; Shehab et al., 2023).

Despite earlier reports suggesting that capsaicin and RTX exert their analgesic effects through nerve degeneration (Jancsó and Lawson, 1990; Pini et al., 1990), other studies reported no changes (Kissin et al., 2007a,b; Oszlács et al., 2015). Furthermore, our recent light microscopy (LM) and electron microscopy (EM) works demonstrated that the perineural application of RTX (0.002 and 0.008%) to the L4 nerve does not induce any damage to the unmyelinated axons of the treated nerve or the corresponding DRG (Javed et al., 2020; Shehab et al., 2023). These results indicate that the analgesic effects of perineural RTX application, which alleviate and prevent nerve injury-induced neuropathic pain, cannot be attributed to nerve degeneration (Javed et al., 2020; Shehab et al., 2023).

**Figure 6:**
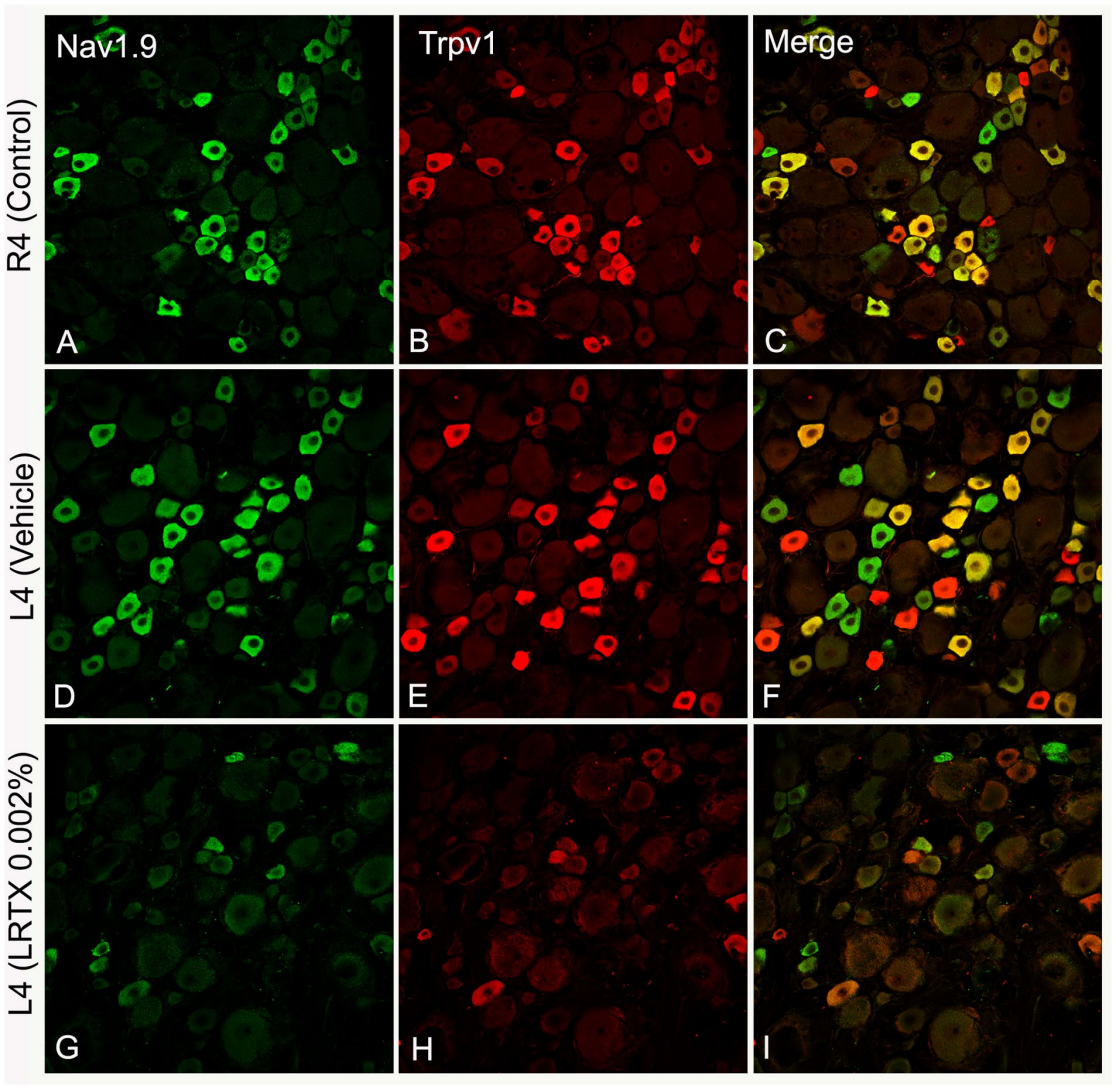
Representative images of double immunofluorescent labeling showing that RTX application on the L4 nerve causes down-regulation of both Nav1.9- **(G)** and Trpv1- **(H)** immunoreactivities in the neurons of the corresponding DRG compared with the right control **(A,B)** and vehicle-treated L4 DRGs **(D,E)**. **(C,F,I)** Are merged images. Scale bar = 50 μm (Shehab et al., 2023).

TRPV1 is a well-characterized heat receptor which is mainly expressed in small-sized sensory neurons in the DRG and unmyelinated peripheral nerves (Caterina et al., 1999; Ichikawa and Sugimoto, 2001; Hironaka et al., 2014; Brown, 2016; Javed et al., 2020). Local application of RTX produced neuroplastic changes in the corresponding DRGs and spinal cord segments, including the downregulation of TRPV1 itself and upregulation of VIP and ATF3 (Javed et al., 2020, 2022). Therefore, the downregulation of TRPV1 following the perineural and cutaneous application of RTX could explain the suppression of thermal hyperalgesia (Javed et al., 2020, 2022). However, the reduction of mechanical hyperalgesia is more likely to be associated with a downregulation of other nociceptive transmitters that colocalize with TRPV1 in the DRG. Indeed, RTX caused the downregulation of both CGRP and IB4 in the DRG (Javed et al., 2020). A follow-up investigation in our laboratory demonstrated that RTX treatment also leads to the downregulation of various other voltage-gated ion channels, including Nav1.9 (Figure 6; Shehab et al., 2023), Kv4.3, and Cav2.2 in the DRG, all of which are associated with pain perception and transmission (Shehab et al., 2023). In contrast, perineural RTX treatment did not alter the levels of Piezo2 or Kv1.1 (markers for mechanoreceptors) or Kir4.1 (a marker for satellite cells) in the DRG (Shehab et al., 2023). Significantly, this downregulation would suppress the excitability of DRG neurons, which is expected to reduce both thermal and mechanical hyperalgesia. Additionally, RTX has been shown to silence its specific receptor (TRPV1) and other relevant neuropeptides and neurotransmitters in the same DRG neurons. This led to the proposal that RTX has a common denominator mechanism of action by which it causes remarkable down-regulation of the TRPV1 receptor as well as many other nociceptive mediators that are mainly found in TRPV1-containing neurons and involved in pain transmission and modulation (Shehab et al., 2023).

### Novel aspects of treating neuropathic pain

1. Using an innovative approach and novel procedures that can completely abolish neuropathic pain in a widely used preclinical animal model have been discovered. To put this in context, studies of human neuropathic pain treatments are considered effective if they produce a 30–50% reduction in pain measures (Attal, 2011).
2. This novel approach abolishes neuropathic pain by treating the uninjured nerves directly adjacent to the injured one responsible for the neuropathy.
3. Low doses of a comparatively inexpensive analog of CAPS, RTX was shown to be 100% effective.
4. The action of RTX is specific to chronic neuropathic pain-neurotransmission in nerves responsible for acute pain, leaving proprioception and touch unaffected.
5. RTX treatment is safe insofar as it does not cause unwanted behavioral deficits or signs of physical damage when administered to uninjured nerves in control animals (without L5 nerve injury and neuropathic pain).

### Translation of critical preclinical results to human patients

#### What is unique about this treatment?

**Figure 7:**
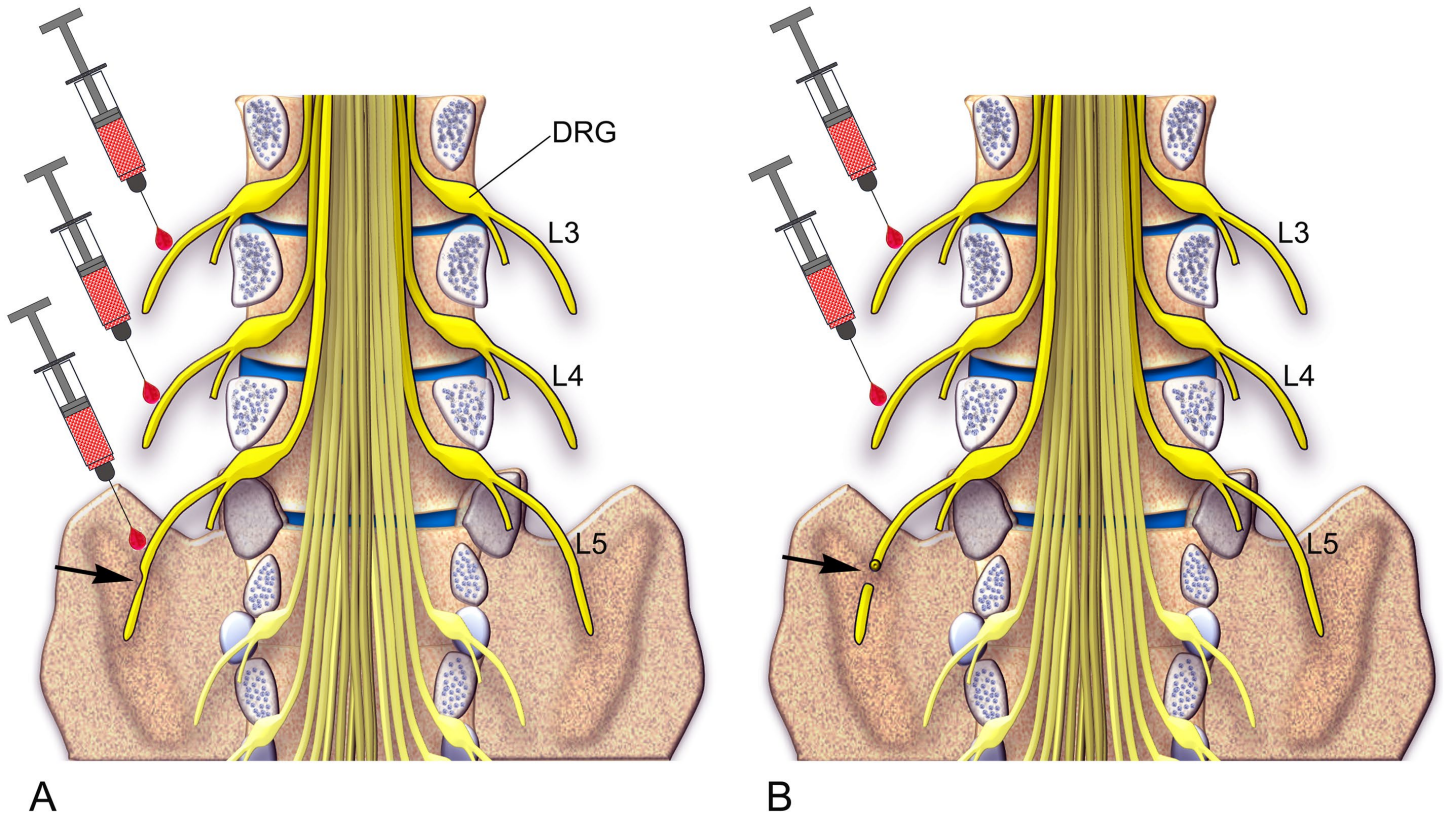
A schematic diagram illustrates two approaches for treating neuropathic pain in human patients based on preclinical findings. **(A)** In Approach 1, for cases where the peripheral nerve is partially damaged, an injection of RTX is recommended into the injured L5 nerve as well as the two uninjured rostral nerves, L3 and L4. **(B)** In Approach 2, for cases where the peripheral nerve is completely damaged, an injection of RTX is recommended into the two uninjured rostral nerves, L3 and L4 (Shehab et al., 2023).

In light of the findings on the treatment of neuropathic pain in animals, a proposal has been put forth to test the feasibility of applying this approach to the treatment of neuropathic pain in humans. The distinctive features of this ambitious translational proposal are as follows:

1. The traditional use treatment of topical creams and patches would be replaced by peri-neuronal injections of RTX.
2. The injections would be targeted at uninjured nerves adjacent to the injury site rather than the area(s) or parts of the body where the pain is felt (Figure 7).
3. The locations of the proposed injection sites are illustrated in Figure 7. Care would be taken to ensure that both nerve fibers and neuronal cell bodies in the DRG were exposed to RTX. The surgical procedures required would be both simple and cheap.
4. Rather than replicate the partially effective treatments that are currently available (30–50% reduction in pain measures), we intend to produce a complete block of neuropathic pain.
5. The pain-specific nature of our treatment means that other sensory and motor functions should remain unaffected.
6. The analgesic effect would be expected to last at least for several months.

Figure 7 summarizes the proposed treatment strategies for neuropathic pain resulting from partial or complete peripheral nerve injury, which may occur due to trauma, surgery, vertebral disc herniation, nerve entrapment, ischemia, or postherpetic lesions. This approach may also be applicable for managing chronic pain associated with diabetic peripheral neuropathy and spinal cord injury. Severe neuropathic pain, a frequent and debilitating outcome of spinal cord injury, remains challenging to treat (Shiao and Lee-Kubli, 2018). Patients with spinal cord injury typically experience pain in a segmental distribution involving one or more dermatomes both above and below the spinal lesion (Bryce et al., 2012). This pattern may be explained by our earlier findings, which suggest that overlapping of the injured and uninjured primary afferents across two spinal segments above and one segment below the lesion plays a key role (Shehab et al., 2008, 2015). Based on this, we propose that perineural injection of RTX into a few peripheral nerves surrounding the spinal lesion could effectively treat neuropathic pain in spinal cord injury patients.

#### Perineural RTX injection in human patients to relieve neuropathic pain

It is essential to determine whether procedures developed in animal models can be successfully translated to the clinic to treat neuropathic pain in human patients, where current treatments have proven ineffective. Several key issues must be considered. First, there are technical challenges in determining the most effective method for administering RTX to the nerves adjacent to the damaged ones. Two strategies will be employed to guide precise RTX injection placement: (i) the use of anatomical landmarks and (ii) supplementation with ultrasound imaging for enhanced accuracy.

#### Detailed therapeutic procedure

Before administering the RTX injection, a thorough sonographic examination will be conducted to locate the injection sites accurately. A diagnostic block using a local anesthetic (0.05–1 mL of 1% ropivacaine) will be applied to the targeted nerves to confirm the scan’s accuracy. The ideal injection site will demonstrate two outcomes: (i) alleviation of the patient’s neuropathic pain and (ii) suppression of any discomfort from the RTX injection itself. Once a suitable target has been identified, a follow-up session will be conducted, during which the appropriate amount of RTX (0.5–1 mL, 0.002%) will be injected, along with the local anesthetic.

#### Assessment of therapeutic advantage

Quantitative measures of neuropathic pain will be assessed using numerical rating scales (Attal et al., 2021), a widely used method where patients rate their subjective pain on a 10-point scale. Following this procedure (Meyer-Frießem et al., 2019), patients will maintain a pain intensity diary, recording their pain at three specific times daily before and after the RTX injection. Additionally, the intensity of spontaneous pain events will be evaluated pre-and post-RTX injection.

Along with these neuropathic pain assessments, detailed measurements of the patient’s acute pain thresholds and their sensitivity to non-noxious tactile stimulation will be taken. Acute pain thresholds will be evaluated using quantitative sensory testing, which measures cold detection threshold (CDT), warm detection threshold (WDT), cold pain threshold (CPT), and heat pain threshold (HPT). Tactile sensitivity will be assessed using von Frey hairs (Meyer-Frießem et al., 2019).

The initial cohort of patients undergoing this treatment will be closely monitored for 5–7 months to track therapeutic outcomes and assess for any side effects. The goal is to demonstrate that this novel treatment can completely suppress neuropathic pain without negatively affecting the perception of acute pain or touch.

#### Patient selection criteria

Inclusion Criteria:

1. Age between 20 and 79 years.
2. Presentation with neuropathic pain due to peripheral nerve injury lasting ≥6 months.
3. History of an unsatisfactory response to conventional and non-conventional treatments for peripheral neuropathic pain.
4. Temporary pain relief following the injection of 1% ropivacaine as a diagnostic measure prior to RTX administration.
5. Use as a preventive measure before surgical procedures to mitigate postsurgical pain in patients with unavoidable peripheral nerve injury.

Exclusion Criteria:

1. Presentation with neuropathic pain affecting major parts of the body bilaterally.
2. Neuropathic pain is caused by factors other than localized peripheral nerve injury, such as infection or chemotherapy-induced neuropathy.

#### Limitations

While the procedure can be performed in an outpatient clinic, patients will need to be admitted to the hospital. Additionally, expertise in ultrasound-guided imaging and injections is essential for the procedure to be carried out effectively.

## Conclusion

Neuropathic pain remains a critical and challenging area within neuroscience due to the intricate nature of its underlying pathology and the inadequacies of current treatments. Emerging data demonstrate that applying capsaicin or its potent analog, resiniferatoxin (RTX), to uninjured nerves adjacent to a damaged nerve provides significant, long-lasting pain relief in preclinical models. Preclinical studies show that localized application of these agents can selectively ablate unmyelinated nociceptive fibers while preserving other sensory functions, leading to substantial, sustained pain relief. These findings indicate that the modulation of peripheral nociceptive inputs represents a promising avenue for the development of novel therapeutic strategies for neuropathic pain.

## Future perspectives

The transition from preclinical efficacy to clinical applicability is an urgent next step. Future research should prioritize rigorous clinical trials to establish the safety, dosage parameters, and therapeutic potential of capsaicin and RTX in diverse neuropathic pain conditions, including diabetic neuropathy, post-surgical pain, and trauma-induced neuropathy. Further investigation into the long-term effects and potential for repeated treatments will be essential to fully understanding and optimizing these therapies. Advancing our understanding of the molecular specificity of capsaicin and RTX on nociceptive fibers could also lead to novel pharmacological derivatives or combined therapies, offering new opportunities to manage neuropathic pain with higher precision and fewer side effects. These endeavors could ultimately transform the therapeutic paradigm, offering robust and potentially curative interventions for neuropathic pain management.

## References

[ref1] Abd-Elsayed A. Vardhan S. Aggarwal A. Vardhan M. Diwan S. A. (2024). Mechanisms of action of dorsal root ganglion stimulation. Int. J. Mol. Sci. 25, 1–17. doi: 10.3390/ijms25073591, PMID: 38612402 PMC11011701

[ref2] Anand P. Bley K. (2011). Topical capsaicin for pain management: therapeutic potential and mechanisms of action of the new high-concentration capsaicin 8% patch. Br. J. Anaesth. 107, 490–502. doi: 10.1093/bja/aer260, PMID: 21852280 PMC3169333

[ref8001] Antunes F. T. T. Huang S. Chen L. Zamponi G. W. (2024). Effect of ABT-639 on Cav3.2 channel activity and its analgesic actions in mouse models of inflammatory and neuropathic pain. Eur. J. Pharmacol. 967. doi: 10.1016/j.ejphar.2024.176416 38342359

[ref8002] Attal N. (2011). Assessment of neuropathic pain in the setting of intervention trials. Clinical Investigation 1, 501–507. doi: 10.4155/CLI.11.22

[ref3] Attal N. Poindessous-Jazat F. De Chauvigny E. Quesada C. Mhalla A. Ayache S. S.. (2021). Repetitive transcranial magnetic stimulation for neuropathic pain: a randomized multicentre sham-controlled trial. Brain 144, 3328–3339. doi: 10.1093/brain/awab208, PMID: 34196698

[ref4] Baron R. Binder A. Wasner G. (2010). Neuropathic pain: diagnosis, pathophysiological mechanisms, and treatment. Lancet Neurol. 9, 807–819. doi: 10.1016/S1474-4422(10)70143-5, PMID: 20650402

[ref5] Bates D. Schultheis B. C. Hanes M. C. Jolly S. M. Chakravarthy K. V. Deer T. R.. (2019). A comprehensive algorithm for management of neuropathic pain. Pain Med. 20, S2–S12. doi: 10.1093/pm/pnz075, PMID: 31152178 PMC6544553

[ref6] Bennett D. L. Clark A. J. Huang J. Waxman S. G. Dib-Hajj S. D. (2019). The role of voltage-gated sodium channels in pain signaling. Physiol. Rev. 99, 1079–1151. doi: 10.1152/physrev.00052.2017, PMID: 30672368

[ref8003] Biogen. (2021). Announces Positive Topline Results from Phase 2 CONVEY Study in Small Fiber Neuropathy. Available at: http://www.biogen.com (Accessed September 16, 2021).

[ref7] Bishnoi M. Bosgraaf C. A. Premkumar L. S. (2011). Preservation of acute pain and efferent functions following intrathecal resiniferatoxin-induced analgesia in rats. J. Pain 12, 991–1003. doi: 10.1016/j.jpain.2011.03.005, PMID: 21680254 PMC3645374

[ref8] Brown D. C. (2016). Resiniferatoxin: the evolution of the "molecular scalpel" for chronic pain relief. Pharmaceuticals (Basel) 9, 1–11. doi: 10.3390/ph9030047, PMID: 27529257 PMC5039500

[ref9] Bryce T. N. Biering-Sørensen F. Finnerup N. B. Cardenas D. D. Defrin R. Lundeberg T.. (2012). International spinal cord injury pain classification: part I. Background and description. March 6-7, 2009. Spinal Cord 50, 413–417. doi: 10.1038/sc.2011.156, PMID: 22182852

[ref10] Campbell J. N. Meyer R. A. (2006). Mechanisms of neuropathic pain. Neuron 52, 77–92. doi: 10.1016/j.neuron.2006.09.021, PMID: 17015228 PMC1810425

[ref11] Caterina M. J. Rosen T. A. Tominaga M. Brake A. J. Julius D. (1999). A capsaicin-receptor homologue with a high threshold for noxious heat. Nature 398, 436–441. doi: 10.1038/18906, PMID: 10201375

[ref12] Catterall W. A. Perez-Reyes E. Snutch T. P. Striessnig J. (2005). International Union of Pharmacology. XLVIII. Nomenclature and structure-function relationships of voltage-gated calcium channels. Pharmacol. Rev. 57, 411–425. doi: 10.1124/pr.57.4.5, PMID: 16382099

[ref13] Charles P. D. (2004). Botulinum neurotoxin serotype a: a clinical update on non-cosmetic uses. Am. J. Health Syst. Pharm. 61, S11–S23. doi: 10.1093/ajhp/61.suppl_6.S11, PMID: 15598005

[ref14] Chien L. Y. Cheng J. K. Chu D. Cheng C. F. Tsaur M. L. (2007). Reduced expression of A-type potassium channels in primary sensory neurons induces mechanical hypersensitivity. J. Neurosci. 27, 9855–9865. doi: 10.1523/JNEUROSCI.0604-07.2007, PMID: 17855600 PMC6672648

[ref15] Cruccu G. Truini A. (2017). A review of neuropathic pain: from guidelines to clinical practice. Pain Ther. 6, 35–42. doi: 10.1007/s40122-017-0087-0, PMID: 29178033 PMC5701894

[ref16] Davis J. B. Gray J. Gunthorpe M. J. Hatcher J. P. Davey P. T. Overend P.. (2000). Vanilloid receptor-1 is essential for inflammatory thermal hyperalgesia. Nature 405, 183–187. doi: 10.1038/35012076, PMID: 10821274

[ref17] De Bruijn T. M. De Groot I. B. Miedema H. S. Haumann J. Ostelo R. W. J. G. (2021). Clinical relevance of epidural steroid injections on lumbosacral radicular syndrome-related Synptoms: systematic review and Meta-analysis. Clin. J. Pain 37, 524–537. doi: 10.1097/AJP.0000000000000943, PMID: 33859113 PMC8162229

[ref8007] Derry S. Moore R. A. (2012). Topical capsaicin (low concentration) for chronic neuropathic pain in adults. Cochrane Database Syst Rev. doi: 10.1002/14651858.CD010111 PMC6540838 22972149

[ref18] Derry S. Rice A. S. Cole P. Tan T. Moore R. A. (2017). Topical capsaicin (high concentration) for chronic neuropathic pain in adults. Cochrane Database Syst. Rev. 1:CD007393. doi: 10.1002/14651858.CD007393.pub4 28085183 PMC6464756

[ref8004] Deuis J. R. Dekan Z. Wingerd J. S. Smith J. J. Munasinghe N. R. Bhola R. F.. (2017). Pharmacological characterisation of the highly Na v 1.7 selective spider venom peptide Pn3a. Sci. Rep. 7. doi: 10.1038/srep40883 PMC5247677 28106092

[ref19] Dib-Hajj S. D. Black J. A. Waxman S. G. (2015). NaV1.9: a sodium channel linked to human pain. Nat. Rev. Neurosci. 16, 511–519. doi: 10.1038/nrn3977, PMID: 26243570

[ref20] Eagles D. A. Chow C. Y. King G. F. (2022). Fifteen years of Na. Br. J. Pharmacol. 179, 3592–3611. doi: 10.1111/bph.15327, PMID: 33206998

[ref21] Fattori V. Hohmann M. S. Rossaneis A. C. Pinho-Ribeiro F. A. Verri W. A. (2016). Capsaicin: current understanding of its mechanisms and therapy of pain and other pre-clinical and clinical uses. Molecules 21, 1–33. doi: 10.3390/molecules21070844, PMID: 27367653 PMC6273101

[ref22] Feng X. J. Ma L. X. Jiao C. Kuang H. X. Zeng F. Zhou X. Y.. (2019). Nerve injury elevates functional Cav3.2 channels in superficial spinal dorsal horn. Mol. Pain 15:1744806919836569. doi: 10.1177/1744806919836569, PMID: 30803310 PMC6458665

[ref8009] Flinspach M. Xu Q. Piekarz A. Fellows R. Hagan R. Gibbs Y.. (2017). Insensitivity to pain induced by a potent selective closed-state Nav. 1.7 inhibitor. Sci. Rep. 7. doi: 10.1038/srep39662 PMC5206724 28045073

[ref23] Finnerup N. B. (2019). Nonnarcotic methods of pain management. N. Engl. J. Med. 380, 2440–2448. doi: 10.1056/NEJMra1807061, PMID: 31216399

[ref24] Finnerup N. B. Attal N. Haroutounian S. Mcnicol E. Baron R. Dworkin R. H.. (2015). Pharmacotherapy for neuropathic pain in adults: a systematic review and meta-analysis. Lancet Neurol. 14, 162–173. doi: 10.1016/S1474-4422(14)70251-0, PMID: 25575710 PMC4493167

[ref25] Finnerup N. B. Kuner R. Jensen T. S. (2021). Neuropathic pain: from mechanisms to treatment. Physiol. Rev. 101, 259–301. doi: 10.1152/physrev.00045.2019, PMID: 32584191

[ref26] Gamse R. (1982). Capsaicin and nociception in the rat and mouse. Possible role of substance P. Naunyn Schmiedeberg's Arch. Pharmacol. 320, 205–216. doi: 10.1007/BF00510129, PMID: 6182473

[ref27] Hironaka K. Ozaki N. Hattori H. Nagamine K. Nakashima H. Ueda M.. (2014). Involvement of glial activation in trigeminal ganglion in a rat model of lower gingival cancer pain. Nagoya J. Med. Sci. 76, 323–332, PMID: 25741041 PMC4345692

[ref28] Ho Kim S. Mo Chung J. (1992). An experimental model for peripheral neuropathy produced by segmental spinal nerve ligation in the rat. Pain 50, 355–363. doi: 10.1016/0304-3959(92)90041-9, PMID: 1333581

[ref29] Horan M. Jacobsen A. H. Scherer C. Rosenlund C. Gulisano H. A. Søe M.. (2021). Complications and effects of dorsal root ganglion stimulation in the treatment of chronic neuropathic pain: a Nationwide cohort study in Denmark. Neuromodulation 24, 729–737. doi: 10.1111/ner.13171, PMID: 32539189

[ref30] Hosomi K. Sugiyama K. Nakamura Y. Shimokawa T. Oshino S. Goto Y.. (2020). A randomized controlled trial of 5 daily sessions and continuous trial of 4 weekly sessions of repetitive transcranial magnetic stimulation for neuropathic pain. Pain 161, 351–360. doi: 10.1097/j.pain.0000000000001712, PMID: 31593002 PMC6970577

[ref31] Iadarola M. J. Brown D. C. Nahama A. Sapio M. R. Mannes A. J. (2021). Pain treatment in the companion canine model to validate rodent results and incentivize the transition to human clinical trials. Front. Pharmacol. 12:705743. doi: 10.3389/fphar.2021.705743, PMID: 34421597 PMC8375595

[ref32] Ichikawa H. Sugimoto T. (2001). VR1-immunoreactive primary sensory neurons in the rat trigeminal ganglion. Brain Res. 890, 184–188. doi: 10.1016/S0006-8993(00)03253-4, PMID: 11164782

[ref33] Iftinca M. C. (2011). Neuronal T-type calcium channels: what's new? Iftinca: T-type channel regulation. J. Med. Life 4, 126–138, PMID: 21776294 PMC3124264

[ref34] Jancsó G. (1992). Pathobiological reactions of C-fibre primary sensory neurones to peripheral nerve injury. Exp. Physiol. 77, 405–431. doi: 10.1113/expphysiol.1992.sp003603, PMID: 1321641

[ref35] Jancśo G. Király E. Jancsó-Gábor A. (1980). Direct evidence for an axonal site of action of capsaicin. Naunyn Schmiedeberg's Arch. Pharmacol. 313, 91–94. doi: 10.1007/BF00505809, PMID: 7207640

[ref36] Jancsó G. Lawson S. N. (1990). Transganglionic degeneration of capsaicin-sensitive C-fiber primary afferent terminals. Neuroscience 39, 501–511. doi: 10.1016/0306-4522(90)90286-D, PMID: 2087270

[ref37] Jang J. H. Kim K. H. Nam T. S. Lee W. T. Park K. A. Kim D. W.. (2007). The role of uninjured C-afferents and injured afferents in the generation of mechanical hypersensitivity after partial peripheral nerve injury in the rat. Exp. Neurol. 204, 288–298. doi: 10.1016/j.expneurol.2006.11.004, PMID: 17184773

[ref8005] Jarvis M. F. Honore P. Shieh C.-C. Chapman M. Joshi S. Zhang X.-F.. (2007). A-803467, a potent and selective Na v 1.8 sodium channel blocker, attenuates neuropathic and inflammatory pain in the rat. Proc. Natl. Acad. Sci. doi: 10.1073/pnas.0611364104 PMC1895982 17483457

[ref38] Javed H. Johnson A. M. Challagandla A. K. Emerald B. S. Shehab S. (2022). Cutaneous injection of Resiniferatoxin completely alleviates and prevents nerve-injury-induced neuropathic pain. Cells 11, 1–23. doi: 10.3390/cells11244049, PMID: 36552812 PMC9776507

[ref39] Javed H. Rehmathulla S. Tariq S. Emerald B. S. Ljubisavljevic M. Shehab S. (2020). Perineural application of resiniferatoxin on uninjured L3 and L4 nerves completely alleviates thermal and mechanical hypersensitivity following L5 nerve injury in rats. J. Comp. Neurol. 528, 2195–2217. doi: 10.1002/cne.24884, PMID: 32064609

[ref40] Joosten E. A. Franken G. (2020). Spinal cord stimulation in chronic neuropathic pain: mechanisms of action, new locations, new paradigms. Pain 161, S104–S113. doi: 10.1097/j.pain.0000000000001854, PMID: 33090743 PMC7434213

[ref41] Kanda H. Ling J. Chang Y. T. Erol F. Viatchenko-Karpinski V. Yamada A.. (2021). Kv4.3 channel dysfunction contributes to trigeminal neuropathic pain manifested with orofacial cold hypersensitivity in rats. J. Neurosci. 41, 2091–2105. doi: 10.1523/JNEUROSCI.2036-20.2021, PMID: 33472822 PMC8018767

[ref8008] Khanna R. Yu J. Yang X. Moutal A. Chefdeville A. Gokhale V.. (2019). Targeting the CaVα-CaVβ interaction yields an antagonist of the N-type CaV2.2 channel with broad antinociceptive efficacy. Pain. 160, 1644–1661. doi: 10.1097/j.pain.0000000000001524 30933958 PMC8998802

[ref42] Kissin I. Freitas C. F. Bradley E. L. (2007a). Perineural resiniferatoxin prevents the development of hyperalgesia produced by loose ligation of the sciatic nerve in rats. Anesth. Analg. 104, 1210–1216, tables of contents. doi: 10.1213/01.ane.0000260296.01813.62, PMID: 17456676

[ref43] Kissin I. Freitas C. F. Mulhern H. L. Degirolami U. (2007b). Sciatic nerve block with resiniferatoxin: an electron microscopic study of unmyelinated fibers in the rat. Anesth. Analg. 105, 825–831. doi: 10.1213/01.ane.0000277491.40055.47, PMID: 17717246

[ref44] Lefaucheur J. P. André-Obadia N. Antal A. Ayache S. S. Baeken C. Benninger D. H.. (2014). Evidence-based guidelines on the therapeutic use of repetitive transcranial magnetic stimulation (rTMS). Clin. Neurophysiol. 125, 2150–2206. doi: 10.1016/j.clinph.2014.05.021, PMID: 25034472

[ref45] Luo Z. D. Chaplan S. R. Higuera E. S. Sorkin L. S. Stauderman K. A. Williams M. E.. (2001). Upregulation of dorsal root ganglion (alpha)2(delta) calcium channel subunit and its correlation with allodynia in spinal nerve-injured rats. J. Neurosci. 21, 1868–1875. doi: 10.1523/JNEUROSCI.21-06-01868.2001, PMID: 11245671 PMC6762626

[ref8010] Lynch S. S. Cheng C. M. Yee J. L. (2006). Intrathecal ziconotide for refractory chronic pain. Ann. Pharmacother. 1293–1300. doi: 10.1345/aph.1G584 16849624

[ref46] Marics I. Malapert P. Reynders A. Gaillard S. Moqrich A. (2014). Acute heat-evoked temperature sensation is impaired but not abolished in mice lacking TRPV1 and TRPV3 channels. PLoS One 9:e99828. doi: 10.1371/journal.pone.0099828, PMID: 24925072 PMC4055713

[ref47] Mcdermott L. A. Weir G. A. Themistocleous A. C. Segerdahl A. R. Blesneac I. Baskozos G.. (2019). Defining the functional role of Na. Neuron 101, 905–919.e8. doi: 10.1016/j.neuron.2019.01.047, PMID: 30795902 PMC6424805

[ref8011] M’Dahoma Gadotti V. M. Zhang F.-X. Park B. Nam J. H. Onnis V. S.. (2016). Effect of the T-type channel blocker KYS-05090S in mouse models of acute and neuropathic pain. Pflugers Archiv Eur. J. Physiol. 468, 193–199. doi: 10.1007/s00424-015-1733-1 26354962

[ref48] Meyer-Frießem C. H. Eitner L. B. Kaisler M. Maier C. Vollert J. Westermann A.. (2019). Perineural injection of botulinum toxin-a in painful peripheral nerve injury - a case series: pain relief, safety, sensory profile and sample size recommendation. Curr. Med. Res. Opin. 35, 1793–1803. doi: 10.1080/03007995.2019.1626228, PMID: 31148462

[ref49] Moran M. M. Szallasi A. (2018). Targeting nociceptive transient receptor potential channels to treat chronic pain: current state of the field. Br. J. Pharmacol. 175, 2185–2203. doi: 10.1111/bph.14044, PMID: 28924972 PMC5980611

[ref50] Mori N. Hosomi K. Nishi A. Miyake A. Yamada T. Matsugi A.. (2024). Repetitive transcranial magnetic stimulation focusing on patients with neuropathic pain in the upper limb: a randomized sham-controlled parallel trial. Sci. Rep. 14:11811. doi: 10.1038/s41598-024-62018-x, PMID: 38782994 PMC11116497

[ref51] Neubert J. K. Mannes A. J. Karai L. J. Jenkins A. C. Zawatski L. Abu-Asab M.. (2008). Perineural resiniferatoxin selectively inhibits inflammatory hyperalgesia. Mol. Pain 4:3. doi: 10.1186/1744-8069-4-3, PMID: 18199335 PMC2242785

[ref52] Ossipov M. H. Porreca F. (2013). Animal models of experimental neuropathic pain, in: McMahon S.B., Koltzenburg M. Tracey I. Turk D. (Eds.) Wall and Melzack's textbook of pain, Elsevier Saunders, 889–901.

[ref53] Oszlács O. Jancsó G. Kis G. Dux M. Sántha P. (2015). Perineural capsaicin induces the uptake and transganglionic transport of choleratoxin B subunit by nociceptive C-fiber primary afferent neurons. Neuroscience 311, 243–252. doi: 10.1016/j.neuroscience.2015.10.042, PMID: 26520849

[ref8013] Patel R. Rutten K. Valdor M. Schiene K. Wigge S. Schunk S.. (2015). Electrophysiological characterization of activation state-dependent Cav2 channel antagonist TROX-1 in spinal nerve injured rats. Neuroscience 297, 47–57. doi: 10.1016/j.neuroscience.2015.03.057 25839150 PMC4436437

[ref8014] Payne C. E. Brown A. R. Theile J. W. Loucif A. J. C. Alexandrou A. J Fuller M. D.. (2015). A novel selective and orally bioavailable Nav1.8 channel blocker, PF-01247324, attenuates nociception and sensory neuron excitability. Br. J. Pharmacol. 172, 2654–2670. doi: 10.1111/bph.13092 25625641 PMC4409913

[ref54] Pini A. Baranowski R. Lynn B. (1990). Long-term reduction in the number of C-fibre nociceptors following capsaicin treatment of a cutaneous nerve in adult rats. Eur. J. Neurosci. 2, 89–97. doi: 10.1111/j.1460-9568.1990.tb00384.x, PMID: 12106106

[ref7001] Pitake S. Middleton L. J. Abdus-Saboor I. Mishra S. K. (2019). Inflammation induced sensory nerve growth and pain hypersensitivity requires the n-type calcium channel cav2.2. Front. Neurosci. 13:1009. doi: 10.3389/fnins.2019.01009 31607850 PMC6761232

[ref8015] Pokkula S. Thakur S. R. (2021). Icariin ameliorates partial sciatic nerve ligation induced neuropathic pain in rats: an evidence of in silico and in vivo studies. J. Pharm. Pharmacol. 73, 874–880. doi: 10.1093/jpp/rgab021 33822115

[ref55] Rasband M. N. Park E. W. Vanderah T. W. Lai J. Porreca F. Trimmer J. S. (2001). Distinct potassium channels on pain-sensing neurons. Proc. Natl. Acad. Sci. USA 98, 13373–13378. doi: 10.1073/pnas.231376298, PMID: 11698689 PMC60878

[ref8016] Rycroft B. K. Vikman K. S. Christie M. J. (2007). Inflammation reduces the contribution of N-type calcium channels to primary afferent synaptic transmission onto NK1 receptor-positive lamina I neurons in the rat dorsal horn. J. Physiol. 580, 883–894. doi: 10.1113/jphysiol.2006.125880 17303639 PMC2075448

[ref56] Sammartino F. Macdonell J. North R. B. Krishna V. Poree L. (2024). Disease applications of spinal cord stimulation: chronic nonmalignant pain. Neurotherapeutics 21:e00314. doi: 10.1016/j.neurot.2023.e00314, PMID: 38184449 PMC11103216

[ref57] Sapio M. R. Neubert J. K. Lapaglia D. M. Maric D. Keller J. M. Raithel S. J.. (2018). Pain control through selective chemo-axotomy of centrally projecting TRPV1+ sensory neurons. J. Clin. Invest. 128, 1657–1670. doi: 10.1172/JCI94331, PMID: 29408808 PMC5873867

[ref58] Shehab S. A. (2014). Fifth lumbar spinal nerve injury causes neurochemical changes in corresponding as well as adjacent spinal segments: a possible mechanism underlying neuropathic pain. J. Chem. Neuroanat. 55, 38–50. doi: 10.1016/j.jchemneu.2013.12.002, PMID: 24394408

[ref59] Shehab S. A. Al-Marashda K. Al-Zahmi A. Abdul-Kareem A. Al-Sultan M. A. (2008). Unmyelinated primary afferents from adjacent spinal nerves intermingle in the spinal dorsal horn: a possible mechanism contributing to neuropathic pain. Brain Res. 1208, 111–119. doi: 10.1016/j.brainres.2008.02.089, PMID: 18395190

[ref60] Shehab S. Anwer M. Galani D. Abdulkarim A. Al-Nuaimi K. Al-Baloushi A.. (2015). Anatomical evidence that the uninjured adjacent L4 nerve plays a significant role in the development of peripheral neuropathic pain after L5 spinal nerve ligation in rats. J. Comp. Neurol. 523, 1731–1747. doi: 10.1002/cne.23750, PMID: 25631932

[ref61] Shehab S. Javed H. Johnson A. M. Tariq S. Kumar C. A. Emerald B. S. (2023). Unveiling the mechanisms of neuropathic pain suppression: perineural resiniferatoxin targets Trpv1 and beyond. Front. Neuroanat. 17:1306180. doi: 10.3389/fnana.2023.1306180, PMID: 38099210 PMC10720729

[ref62] Shehab S. A. Spike R. C. Todd A. J. (2004). Do central terminals of intact myelinated primary afferents sprout into the superficial dorsal horn of rat spinal cord after injury to a neighboring peripheral nerve? J. Comp. Neurol. 474, 427–437. doi: 10.1002/cne.20147, PMID: 15174085

[ref63] Shiao R. Lee-Kubli C. A. (2018). Neuropathic pain after spinal cord injury: challenges and research perspectives. Neurotherapeutics 15, 635–653. doi: 10.1007/s13311-018-0633-4, PMID: 29736857 PMC6095789

[ref64] Shinu P. Morsy M. A. Nair A. B. Mouslem A. K. A. Venugopala K. N. Goyal M.. (2022). Novel therapies for the treatment of neuropathic pain: potential and pitfalls. J. Clin. Med. 11, 1–26. doi: 10.3390/jcm11113002, PMID: 35683390 PMC9181614

[ref65] Simopoulos T. T. Kraemer J. Nagda J. V. Aner M. Bajwa Z. H. (2008). Response to pulsed and continuous radiofrequency lesioning of the dorsal root ganglion and segmental nerves in patients with chronic lumbar radicular pain. Pain Physician 11, 137–144. doi: 10.36076/ppj.2008/11/137, PMID: 18354708

[ref66] Smith P. A. (2020). K+ channels in primary afferents and their role in nerve injury-induced pain. Front. Cell. Neurosci. 14:566418. doi: 10.3389/fncel.2020.566418, PMID: 33093824 PMC7528628

[ref67] Smith P. A. (2023). Neuropathic pain; what we know and what we should do about it. Front Pain Res 4:1220034. doi: 10.3389/fpain.2023.1220034, PMID: 37810432 PMC10559888

[ref68] Stevens E. B. Stephens G. J. (2018). Recent advances in targeting ion channels to treat chronic pain. Br. J. Pharmacol. 175, 2133–2137. doi: 10.1111/bph.14215, PMID: 29878335 PMC5980455

[ref69] Szigeti C. Sántha P. Körtvély E. Nyári T. Horváth V. J. Deák É.. (2012). Disparate changes in the expression of transient receptor potential vanilloid type 1 receptor mRNA and protein in dorsal root ganglion neurons following local capsaicin treatment of the sciatic nerve in the rat. Neuroscience 201, 320–330. doi: 10.1016/j.neuroscience.2011.10.058, PMID: 22108615

[ref70] Szolcsányi J. (2014). Capsaicin and sensory neurones: a historical perspective. Prog. Drug Res. 68, 1–37 doi: 10.1007/978-3-0348-0828-6_1, PMID: 24941663

[ref71] Takahashi Y. Chiba T. Sameda H. Ohtori S. Kurokawa M. Moriya H. (2002). Organization of cutaneous ventrodorsal and rostrocaudal axial lines in the rat hindlimb and trunk in the dorsal horn of the spinal cord. J. Comp. Neurol. 445, 133–144. doi: 10.1002/cne.10158, PMID: 11891658

[ref72] Takahashi Y. Nakajima Y. Sakamoto T. (1994). Dermatome mapping in the rat hindlimb by electrical stimulation of the spinal nerves. Neurosci. Lett. 168, 85–88. doi: 10.1016/0304-3940(94)90422-7, PMID: 7518069

[ref73] Todd A. J. (2010). Neuronal circuitry for pain processing in the dorsal horn. Nat. Rev. Neurosci. 11, 823–836. doi: 10.1038/nrn2947, PMID: 21068766 PMC3277941

[ref74] Tsantoulas C. Mcmahon S. B. (2014). Opening paths to novel analgesics: the role of potassium channels in chronic pain. Trends Neurosci. 37, 146–158. doi: 10.1016/j.tins.2013.12.002, PMID: 24461875 PMC3945816

[ref75] Tsantoulas C. Zhu L. Shaifta Y. Grist J. Ward J. P. Raouf R.. (2012). Sensory neuron downregulation of the Kv9.1 potassium channel subunit mediates neuropathic pain following nerve injury. J. Neurosci. 32, 17502–17513. doi: 10.1523/JNEUROSCI.3561-12.2012, PMID: 23197740 PMC3713313

[ref76] Van Velzen M. Dahan A. Niesters M. (2020). Neuropathic pain: challenges and opportunities. Front. Pain. Res. 1:1. doi: 10.3389/fpain.2020.00001, PMID: 35295693 PMC8915755

[ref77] Walsh T. Malhotra R. Sharma M. (2022). Radiofrequency techniques for chronic pain. BJA Educ. 22, 474–483. doi: 10.1016/j.bjae.2022.08.004, PMID: 36406037 PMC9669778

[ref8017] Wang Y. Zhu D. Ortiz-Velez L. C. Beeton C. (2023). A bioengineered probiotic for the oral delivery of a peptide Kv1.3 channel blocker to treat rheumatoid arthritis. Proc. Proc. Natl. Acad. Sci. e2211977120. doi: 10.1073/pnas.2211977120 PMC9926172 36595694

[ref78] Waxman S. G. Zamponi G. W. (2014). Regulating excitability of peripheral afferents: emerging ion channel targets. Nat. Neurosci. 17, 153–163. doi: 10.1038/nn.3602, PMID: 24473263

[ref79] Woodbury C. J. Zwick M. Wang S. Lawson J. J. Caterina M. J. Koltzenburg M.. (2004). Nociceptors lacking TRPV1 and TRPV2 have normal heat responses. J. Neurosci. 24, 6410–6415. doi: 10.1523/JNEUROSCI.1421-04.2004, PMID: 15254097 PMC6729548

[ref80] Yaksh T. L. (2006). Calcium channels as therapeutic targets in neuropathic pain. J. Pain 7, S13–S30. doi: 10.1016/j.jpain.2005.09.007, PMID: 16426997

[ref8018] Yang F. Zou Y. Q. Li M. Luo W. J. Chen G. Z. Wu X. Z. (2021). Intervertebral foramen injection of plerixafor attenuates neuropathic pain after chronic compression of the dorsal root ganglion: Possible involvement of the down-regulation of Nav1.8 and Nav1.9. Eur. J. Pharmcol. 908:174322. doi: 10.1016/j.ejphar.2021.174322 34256084

[ref8019] Yuan X. Han S. Manyande A. Gao F. Wang J. Zhang W.. (2023). Spinal voltage-gated potassium channel Kv1.3 contributes to neuropathic pain via the promotion of microglial M1 polarization and activation of the NLRP3 inflammasome. Eur. J. Pain. 27, 289–302. doi: 10.1002/ejp.2059” 36440534

[ref81] Zhang J. Zhang R. Wang Y. Dang X. (2024). Efficacy of epidural steroid injection in the treatment of sciatica secondary to lumbar disc herniation: a systematic review and meta-analysis. Front. Neurol. 15:1406504. doi: 10.3389/fneur.2024.1406504, PMID: 38841695 PMC11150834

